# Prospective Associations Between Maternal Depression and Infant Sleep in Women With Gestational Diabetes Mellitus

**DOI:** 10.3389/fpsyg.2022.926315

**Published:** 2022-06-13

**Authors:** Leah Gilbert, Vania Sandoz, Dan Yedu Quansah, Jardena J. Puder, Antje Horsch

**Affiliations:** ^1^Department Woman-Mother-Child, Interdisciplinary GDM Group Lausanne, Obstetric Service, Lausanne University Hospital, Lausanne, Switzerland; ^2^Faculty of Biology and Medicine, Lausanne Perinatal Research Group, Institute of Higher Education and Research in Healthcare, University of Lausanne, Lausanne, Switzerland; ^3^Department Woman-Mother-Child, Child Abuse and Neglect Team, Lausanne University Hospital, Lausanne, Switzerland; ^4^Neonatalogy Unit, Department Woman-Mother-Child, Lausanne University Hospital, Lausanne, Switzerland

**Keywords:** EPDS, BISQ, night waking, obesity, postpartum, mother, overweight

## Abstract

**Background:**

Women with gestational diabetes mellitus have higher rates of perinatal depressive symptoms, compared to healthy pregnant women. In the general population, maternal depressive symptoms have been associated with infant sleep difficulties during the first year postpartum. However, there is lack of data on infants of mothers with gestational diabetes mellitus.

**Methods:**

This study assessed the prospective associations between maternal perinatal depressive symptoms and infant sleep outcomes. The study population consisted of 95 Swiss women with gestational diabetes mellitus and their infants, enrolled in the control group of the *MySweetheart trial* (NCT02890693). Perinatal depressive symptoms were assessed with the Edinburgh Postnatal Depression Scale at the first gestational diabetes mellitus visit during pregnancy, at 6–8 weeks postpartum, and 1 year postpartum. The Brief Infant Sleep Questionnaire was used to assess infant sleep (i.e., nocturnal sleep duration, number of night waking, and maternal perception of infant sleep) at 1 year postpartum. Relevant maternal and infant measurements (e.g., infant sex or maternal age or social support) were collected or extracted from medical records as covariates.

**Results:**

Antenatal maternal depressive symptoms at the first gestational diabetes mellitus visit were inversely associated with infant nocturnal sleep duration at 1 year postpartum (β = –5.9, *p* = 0.046). This association became marginally significant when covariates were added (β = –5.3, *p* = 0.057). Maternal depressive symptoms at 6–8 weeks postpartum were negatively and prospectively associated with infant nocturnal sleep duration (β = –9.35, *p* = 0.016), even when controlling for covariates (β = –7.32, *p* = 0.042). The association between maternal depressive symptoms and maternal perception of infant sleep as *not a problem at all* was significant at 1 year postpartum (β = –0.05, *p* = 0.006), although it became non-significant when controlling for appropriate covariates. No other significant associations were found.

**Limitations:**

This study solely included measures derived from self-report validated questionnaires.

**Conclusion:**

Our findings suggest it is of utmost importance to support women with gestational diabetes mellitus as a means to reduce the detrimental impact of maternal perinatal depressive symptoms on infant sleep, given its predictive role on infant metabolic health.

## Introduction

Gestational diabetes mellitus (GDM) is a metabolic disorder characterised by a glucose intolerance diagnosed between 24 and 28 weeks of gestation (American Diabetes Association, 2022) and affects up to 11.8% of Swiss pregnant women (Ryser Rüetschi et al., 2016). There is a close link between GDM and mental health. Depressive symptoms in early pregnancy are associated with the development of GDM and, in turn, GDM is a risk factor for perinatal depression (Hinkle et al., 2016). Depression is defined by a general state of low mood and/or anhedonia (American Psychiatric Association, 2013). While 13% of pregnant women in the general population develop an episode of major depression (Gavin et al., 2005), up to 28% of women with GDM experience severe symptoms of depression during pregnancy (Wilson et al., 2020). Compared to healthy pregnancies, the risk is 4.6-fold increased in the postpartum (Hinkle et al., 2016).

In the general population, perinatal maternal depression has negative consequences on various child health outcomes, such as emotional development or sleep (Slomian et al., 2019). A growing body of evidence has shown associations between perinatal maternal depression and infant sleep (Armitage et al., 2009; Slomian et al., 2019; Castro Dias and Figueiredo, 2021). A recent study observed prospective unidirectional associations between maternal antenatal depressive symptoms and future infant sleep problems, whereas in the postpartum period, bidirectional associations were found between maternal depressive symptoms and infant sleep problems (Castro Dias and Figueiredo, 2021). To the best of our knowledge, no study has yet investigated the prospective associations between maternal perinatal symptoms of depression and infant sleep in the GDM population.

At age one, Swiss guidelines recommend infants to sleep between 11 and 14 h over a period of 24 h (Association Vaudoise d’Aide et de Soins à Domicile, 2019). Given the important role of nocturnal sleep on overall infant health, Swiss health experts also provided a specific recommendation for nocturnal sleep; that infants sleep between 10 and 12 h per night (Naître et grandir, 2019). A Swiss cohort study also observed a mean of 11.7 h of nocturnal sleep in one-year-old infants (Iglowstein et al., 2003). In addition, short nocturnal sleep duration and infant night waking are typically regarded by mothers as sleep problems (Byars et al., 2012; Williamson et al., 2019). Given the relationships between poor sleep and adverse metabolic outcomes (e.g., childhood obesity and overweight) (Miller et al., 2018), it is of utmost importance to understand the mechanisms that may alter an adequate sleep acquisition in infants. This is especially important in GDM populations, as their offspring are already at a higher risk of childhood obesity (Zhao et al., 2016).

The current study therefore investigated the prospective associations between maternal depressive symptoms during the third trimester of pregnancy and up to 1 year postpartum and infant sleep at 1 year of age in the context of GDM. We hypothesised inverse prospective associations between maternal depressive symptoms and infant nocturnal sleep duration, as well as with maternal perception of infant sleep as being not a problem at all. Inversely, we expected positive associations between maternal depressive symptoms and the number of infant night waking. Considering the aforementioned evidence and our hypothesis, this prospective cohort study of women with GDM investigated the associations between maternal depressive symptoms, (American Diabetes Association, 2022) at first GDM visit (24–32 weeks of gestation), (Ryser Rüetschi et al., 2016) at 6–8 weeks postpartum, and (Hinkle et al., 2016) at 1 year postpartum with infant sleep at 1 year postpartum (infant nocturnal sleep duration, number of night waking, and maternal perception of infant sleep). In addition, this study addressed these associations by controlling for important and relevant covariates related to the mother and the infant.

## Materials and Methods

### Setting and Patient Population

This prospective clinical cohort study included pregnant women diagnosed with GDM according to the International Association of the Diabetes and Pregnancy Study Groups and American Diabetes Association guidelines (American Diabetes Association, 2021), who were taking part in the *MySweetheart trial* (NCT02890693; Horsch et al., 2018). Data collection occurred at the GDM clinic of a Swiss university hospital between September 2016 and October 2021. The Human Research Ethics Committee of the Canton de Vaud approved the study protocol (study number 2016-00745).

### Inclusion and Exclusion Criteria

All women included in this study took part in the *MySweetheart trial.* Thus, these women had a confirmation of GDM diagnosis between 24 and 32 weeks of gestation, were 18 years or older and understood English or French (Horsch et al., 2018). This study included the women that were a part of the control group of the *MySweetheart trial* (*N* = 106 of the total 211 women participating in the *MySweetheart trial*). We chose to only include women of the control group, as women from the intervention group were offered an interdisciplinary lifestyle and psychosocial intervention which aimed at improving mental health in mothers and infant behaviours (including sleep) and thus, would have likely influenced the outcomes of the study at hand. The details of the *MySweetheart trial* have been previously described (Horsch et al., 2018). Briefly, it is a randomized controlled trial with an intervention group receiving a multidimensional interdisciplinary lifestyle and psychosocial intervention aimed at improving mental and metabolic health in women with GDM and their offspring. The control group of the *MySweetheart trial*, analysed for the purpose of this study, received usual care (Horsch et al., 2018). All women provided a signed informed consent. We excluded women who were lost to follow-up (*N* = 11), for the following reasons: moving to different cities, being too busy or not answering our calls at the 6–8 week postpartum visit (*N* = 3) and at the one-year postpartum visit (*N* = 8). Thus, 95 women were included in the current analysis.

### Gestational Diabetes Mellitus Management/Clinical Care

All participants underwent the usual care for the management of their GDM (American Diabetes Association, 2022). At the first clinical visit after the confirmation of GDM diagnosis, women received information on GDM from a nurse or medical doctor specialized in GDM. After this first visit, the *MySweetheart trial* was presented to eligible women. A few days later, women were called to follow-up the initial information and to seek signed informed consent. A week after their first clinical visit, women were seen by a dietician and received recommendations regarding eating habits and weight gain in pregnancy (Blumer et al., 2013). For study participants, the first assessment involved measuring maternal cardio-metabolic and mental health outcomes using standardised testing procedures and self-report questionnaires [(Horsch et al., 2018) for more details]. At 6–8 weeks and at 1 year postpartum, these women underwent an oral glucose tolerance test (Blumer et al., 2013), we assessed cardio-metabolic outcomes, and they completed self-report questionnaires that evaluated their mental health (Horsch et al., 2018).

### Measures

#### Maternal Mental Health

*Edinburgh Postnatal Depression Scale (EPDS)*: Depressive symptoms in the preceding 7 days were assessed at the first GDM visit, 6–8 weeks, and 1 year postpartum (Cox et al., 1987). Each item of this self-report questionnaire is scored on a 4-point scale, the minimum and maximum total scores being 0 and 30, respectively, with higher scores indicating greater symptom severity (Cox et al., 1987) and scores ≥ 11 usually indicating clinically relevant depression scores (Bunevicius et al., 2009). The EPDS has been validated in pregnant women (Bunevicius et al., 2009) and in a French-speaking sample (Guedeney and Fermanian, 1998). Good psychometric properties have been reported (Guedeney and Fermanian, 1998). The Cronbach alpha was moderate to high for each time-point with 0.77 for the first GDM visit, 0.81 for 6–8 weeks postpartum and 0.71 at 1 year postpartum.

#### Infant Sleep Outcomes

*Brief Infant Sleep Questionnaire (BISQ):* This maternal-report questionnaire was used to assess infant sleep variables at 1 year postpartum over the previous week, such as nocturnal sleep duration (between 7 pm and 7 am), number of night waking, and maternal perception of infant sleep with answers ranging from 1: “*A very serious problem”* to 3: *“Not a problem at all,”* these three variables were treated as continuous outcomes (Ma et al., 2022). Furthermore, infant birth order (oldest, middle, youngest) and infant sleep arrangements (whether the infant is alone in a room or sleeps with someone else) were noted (Sadeh, 2004). A French translation and cultural adaptation was carried out according to the forward–backward method (Wild et al., 2005), and this questionnaire demonstrated good psychometric properties (Sadeh, 2004).

Given the Swiss sleep recommendations for one-year-old infants (Association Vaudoise d’Aide et de Soins à Domicile, 2019) and the actual mean observed nocturnal sleep duration of 11.7 h (Iglowstein et al., 2003), we chose a conservative threshold of 11 h as being the sleep nocturnal duration cut-off for the *post hoc* analysis. Thus, we categorised infant nocturnal sleep duration into two types: infants sleeping ≤ 11 *vs.* > 11 h. In addition, maternal perception of infant sleep was classified into “*sleep is not a problem at all”* vs. *“sleep is a small to very serious problem.”*

#### Maternal and Infant Covariate Measurements

At the first GDM visit, maternal age, weeks of gestation, prior GDM diagnosis, educational level, ethnicity, parity, and gravidity were collected or extracted from medical records. Furthermore, glycated haemoglobin (HbA1c) was measured using a chemical photometric method (conjugation with boronate; Afinion^®^) (Jeppsson et al., 2002). In addition, infant weight, size, and sex were extracted from medical records at birth. Measures of infant weight and size were repeated at the 6–8 weeks postpartum visit and at 1 year postpartum. Birth weight percentiles were calculated according to the Intergrowth-21 Standards and Tools (Intergrowth, 2021). BMI percentiles at 6–8 weeks and 1 year postpartum were calculated based on the World Health Organisation child growth standards (WHO Child Growth Standards, 2008). Infant BMI category (slim to normal, overweight or obese), with infants considered slim to normal weight when their BMI percentile was ≤ 84.99th and considered overweight when their BMI percentile was between 85 and 96.99th, and considered obese when their BMI percentile was ≥ 97th at 6–8 weeks and 1 year postpartum (World Health Organization, 2022). Factors related to maternal mental health and infant sleep were also examined as potential covariates. For the sake of brevity, these variables are briefly described hereafter, and additional details can be found in Horsch et al. (2018). Maternal sleep was assessed by the Pittsburgh Sleep Quality Index (PSQI) at the first GDM visit. Maternal anxiety [measured with the Hospital Anxiety and Depression Scale (HADS-A)] and social support [assessed by the Medical Outcomes Study Social Support Survey-short form (mMOS-SS)] were collected at the first GDM visit and at 1 year postpartum.

### Data Analysis

All analyses were carried out with R studio version 1.4.1106 and R version 2.7-1. Descriptive analyses were conducted for socio-demographic variables (Table 1). Continuous and normally distributed variables were described as means and standard deviations and ordinal outcomes were described as frequencies and percentages. Statistical significance was set at *p* < 0.05 as a one-sided test.

**TABLE 1:** Descriptive sample characteristics.

Variable	Participants (*n* = 95)	
	*M* (*SD*)	*n* (%)
Maternal age, *n* = 95	32.38 (4.79)	
Gestational age at birth [days], *n* = 94	273.95 (12.37)	
HbA1c at first GDM visit [nmol], *n* = 92	5.25 (0.36)	
Previous GDM, *n* = 45		10 (22.22)
**Education, *n* = 85**		
Compulsory education incomplete or complete		11 (12.94)
Secondary education (high school)		7 (8.24)
Apprenticeship		19 (22.35)
Higher education (e.g., University)		48 (56.47)
**Ethnicity, *n* = 86**		
Swiss		28 (32.56)
European		37 (43.02)
Non-European		21 (24.42)
**Parity, *n* = 95**		
0		59 (62.11)
1		23 (24.21)
2–4		13 (13.68)
Infant sex [girls], *n* = 95		48 (50.5)
Breastfeeding at 6–8 weeks postpartum [yes], *n* = 89		74 (83.15)
Breastfeeding at 1 year postpartum [yes], *n* = 87		21 (24.14)
Weight at birth [kgs], *n* = 94	3.26 (0.53)	
Z-scores at birth, *n* = 94	0.2 (1)	
Weight at 6–8 weeks postpartum [kgs], *n* = 93	4.65 (0.76)	
Z-scores at 6–8 weeks postpartum, *n* = 94	–0.28 (2.07)	
Weight at 1 year postpartum [kgs], *n* = 94	9.74 (1.16)	
Z-scores at 1 year postpartum, *n* = 94	0.16 (1.08)	
EPDS total score at first GDM visit, *n* = 94	7.35 (4.5)	
Clinically relevant depressive symptoms at first GDM visit*		25 (26.6)
EPDS total score at 6–8 weeks postpartum, *n* = 94	5.75 (3.89)	
Clinically relevant depressive symptoms at 6–8 weeks postpartum*		13 (13.83)
EPDS total score at 1 year postpartum, *n* = 94	5.86 (3.65)	
Clinically relevant depressive symptoms at 1 year postpartum*		13 (13.83)
Nocturnal infant sleep duration at 1 year postpartum [min], *n* = 74	630.16 (135.5)	
Infants sleeping > 660 min*		36 (48.65)
Infants sleeping ≤ 660 min*		38 (51.35)
Number of infant night waking at 1 year postpartum, *n* = 85	1.31 (1.3)	
Maternal perception of infant sleep, *n* = 86		
A very serious problem		5 (5.81)
A small problem		25 (29.07)
Not a problem at all		56 (65.12)

EPDS, Edinburgh Postnatal Depression Scale; HADS-A, anxiety of the Hospital Anxiety and Depression Scale; GDM, gestational diabetes mellitus; mMOS-SS, Medical Outcomes Study Social Support Survey-short form. *We used a cut off of ≥ 11 to indicate women with clinically relevant depressive symptoms for all time points.

We first investigated the prospective associations between maternal depression scores at the first GDM visit and the subsequent infant sleep outcomes using linear regression analyses with infant nocturnal sleep duration (min), number of night waking, and maternal perception of infant sleep, all at 1 year postpartum, as the dependent variables. For the second and third objective, the predictors were maternal depression scores at 6–8 weeks postpartum and at 1 year postpartum, while infant sleep outcomes remained the same.

For all linear regressions, two models (model 1 and 2) were tested. Model 1 was not adjusted for any covariates, while in model 2, variables that were significantly correlated with the respective dependent variable were added as covariates. The following potential covariates were tested via Pearson’s correlations: gestational age at birth, infant nocturnal sleep duration, number of infant night waking, maternal perception of infant sleep, infant birth order, infant weight at birth, at 6–8 weeks and 1 year postpartum, infant weight percentile at birth, infant BMI category, infant sex, maternal age, and HADS-A at first GDM visit and at 1 year postpartum, PSQI at the first GDM visit, and mMOS-SS at the first GDM visit and at 1 year postpartum. For the outcome *infant nocturnal sleep duration*, number of infant night waking (*r* = –0.23, *p* = 0.045) and maternal perception of infant sleep (*r* = 0.36, *p* = 0.001) were used as covariates. For the outcome *number of infant night waking*, infant nocturnal sleep duration (*r* = –0.23, *p* = 0.045), maternal perception of infant sleep (*r* = – 0.61, *p* ≤ 0.001), infant sleep arrangements (*r* = 0.26, *p* = 0.017), maternal age (*r* = 0.24, *p* = 0.026), and maternal mMOS score at the first GDM visit (*r* = –0.38, *p* = 0.007) and at 1 year postpartum (*r* = –0.22, *p* = 0.04) were added as covariates. Finally, for the outcome of *maternal perception of infant sleep*, infant nocturnal sleep duration (*r* = 0.36, *p* = 0.001), number of infant night waking (*r* = –0.61, *p* ≤ 0.001), maternal HADS-A score at 1 year postpartum (*r* = –0.22, *p* = 0.043), and infant sex (*r* = 0.28, *p* = 0.009) were added as covariates. For each model, *post hoc* power analyses were conducted. The range of *post hoc* power of significant associations is between 0.51 and 0.75, and 0.96 for the adjusted model. For the non-significant associations, *post hoc* power indices range from 0.16 to 1.

## Results

### Descriptive Results

As shown in Table 1, most of the study participants were Swiss (32.56%) or European (43.02%). In addition, a quarter of participants (26.6%) reported clinically relevant depressive symptoms at the first GDM visit, while this percentage decreased to 13.83% at 6–8 weeks and 1 year postpartum. Approximately one-third of mothers (34.88%) perceived their infant’s sleep as problematic. Finally, 51.35% of the infants in our sample slept ≤ 11 h (630.16 ± 135.5 min), respectively.

### Prospective Associations Between Maternal Depressive Symptoms at First Gestational Diabetes Mellitus Visit and Infant Sleep at 1 Year

There was a significant inverse prospective association between mean EPDS total score at the first GDM visit and infant nocturnal sleep duration at 1 year postpartum (β = –5.9, *CI 95%* [–12.772, 0.979], *p* = 0.046, Table 2). Conversely, no associations were found between mean EPDS total scores at the first GDM visit and the number of infant night waking or maternal perception of infant sleep (all *p* ≥ 0.05). The association between mean EPDS total score at the first GDM visit and infant nocturnal sleep duration at 1 year postpartum remained marginally significant after adjusting for covariates (β = –5.3, *CI 95%* [–11.904, 1.301], *p* = 0.057). No other significant model emerged.

**TABLE 2:** Associations between maternal depressive symptoms at first GDM visit, 6–8 weeks postpartum and 1 year postpartum with infant sleep characteristics at 1 year postpartum before (model 1) and after adjustments (model 2).

Predictor	Dependent variable	Model 1				Model 2			
		*n*	β	*95% CI*	*P*	*n*	β	*95% CI*	*p*
Total EPDS score at first GDM visit	Nocturnal sleep duration	74	–5.9	[–12.772, 0.979]	**0.046**	74	–5.3	[–11.904, 1.301]	0.057
Total EPDS score at first GDM visit	Number of night waking	84	0.02	[–0.039, 0.088]	0.222	84	–0.02	[–0.1, 0.053]	0.735
Total EPDS score at first GDM visit	Maternal perception of infant sleep	85	–0.01	[–0.039, 0.019]	0.247	85	0	[–0.453, 0.006]	0.404
Total EPDS score at 6–8 weeks postpartum	Nocturnal sleep duration	73	–9.35	[–17.937, –0.762]	**0.017**	73	–7.32	[–15.672, –1.032]	**0.042**
Total EPDS score at 6–8 weeks postpartum	Number of night waking	84	0.04	[–0.036, 0.114]	0.155	84	–0.04	[–0.127, 0.047]	0.822
EPDS at 6–8 weeks postpartum	Maternal perception of infant sleep	85	–0.03	[–0.062, 0.006]	0.053	85	0	[–0.034, 0.034]	0.498
Total EPDS score at 1 year postpartum	Nocturnal sleep duration	73	–4.14	[–12.853, 4.574]	0.173	73	–0.46	[–9.179, 8.259]	0.458
Total EPDS score at 1 year postpartum	Number of night waking	84	0.05	[–0.031, 0.132]	0.11	84	0.01	[–0.072, 0.098]	0.378
Total EPDS score at 1 year postpartum	Maternal perception of infant sleep	85	–0.05	[–0.083, –0.011]	**0.007**	85	–0.02	[–0.056, 0.011]	0.094

*EPDS, Edinburgh Postnatal Depression Scale; GDM, gestational diabetes mellitus. In model 2 the following covariates were added: number of infant night waking and maternal perception of infant sleep for the outcome infant nocturnal sleep duration; infant nocturnal sleep duration, maternal perception of infant sleep, infant sleep arrangements, maternal age, and maternal mMOS-SS score at the first GDM visit and at 1 year postpartum for the outcome number of infant night waking; and infant nocturnal sleep duration, number of infant night waking, maternal HADS-A score at 1 year postpartum, and infant sex for the outcome of maternal perception of infant sleep. P-values smaller than 0.05 are highlighted in bold.*

### Prospective Associations Between Maternal Depressive Symptoms at 6–8 Weeks Postpartum and Infant Sleep at 1 Year

The mean EPDS total score assessed at 6–8 weeks postpartum was also negatively associated with later infant nocturnal sleep duration (β = –9.35, *CI 95%* [–17.937, –0.762], *p* = 0.017, Table 2). Results also showed a marginally significant prospective and negative association between mean EPDS total score at 6–8 weeks postpartum and mothers considering infant sleep as more problematic (β = –0.03, *CI 95%* [–0.062, 0.006], *p* = 0.053), but not with number of infant waking. Only the inverse association between mean EPDS total score and infant nocturnal sleep duration remained significant after controlling for the appropriate covariates (β = –7.32, *CI 95%* [–15.672, 1.032], *p* = 0.042; Table 2).

### Associations Between Maternal Depressive Symptoms at 1 Year Postpartum and Infant Sleep at 1 Year

Mean EPDS total score at 1 year postpartum was negatively associated with maternal perception of infant sleep (β = –0.05, *CI 95%* [–0.083, –0.011], *p* = 0.006, Table 2), while no significant association was observed between mean EPDS total score and infant nocturnal sleep duration or number of night waking. When controlling for relevant covariates, the negative association between mean EPDS total score and maternal perception of infant sleep became non-significant (β = –0.02, *CI 95%* [–0.056, 0.011], *p* = 0.094).

### *Post hoc* Analysis

**FIGURE 1:**
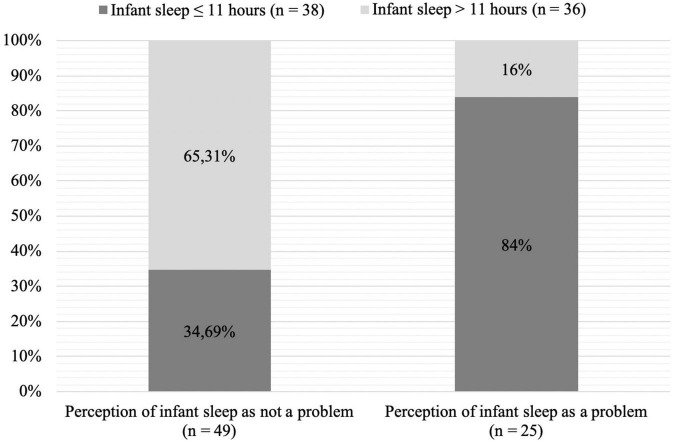
Chart of maternal perception of infant sleep by amount of infant nocturnal sleep (*χ2* (1, 74) = 16.11, *p* < 0.001).

Given that 51.35% of our infant sample slept ≤ 11 h (i.e., 630.16 ± SD 135.5 min) and that only one-third of mothers considered their infant sleep as problematic, we conducted a *post hoc* analysis investigating the associations between the type of infant sleepers and maternal perception of infant sleep as problematic. There was a significant relationship between maternal perception of infant sleep and type of infant sleepers (*χ2* (1, 74) = 16.11, *p* < 0.001). Nonetheless, mothers have a good prediction of their infant sleep when it is ≥ 11 h as they usually perceive the infant’s sleep as not being a problem in most of the cases, but they have ½ chance to perceive their infant sleep as not a problem, when the infant actually sleeps ≤ 11 h. Proportions are displayed in Figure 1.

## Discussion

To our knowledge, this prospective clinical cohort study investigated for the first time the associations between maternal depressive symptoms and infant sleep outcomes within a GDM population. Maternal depressive symptoms, during pregnancy were negatively and prospectively associated with infant nocturnal sleep duration at 1 year postpartum. In addition, maternal depressive symptoms at 6–8 weeks postpartum were negatively and prospectively associated with infant nocturnal sleep duration at 1-year postpartum. Finally, an inverse association between maternal depressive symptoms and maternal perception of infant sleep assessed at 1 year postpartum was observed. As part of a *post hoc* analysis, we found a significant association between maternal perception of infant sleep and infant nocturnal sleep duration.

The results showing prospective associations between maternal depressive symptoms at first GDM visit and shorter infant sleep duration at 1 year postpartum in this cohort partially echoes previous findings in healthy pregnant women (Castro Dias and Figueiredo, 2021; Ma et al., 2022). A recent prospective study also found a negative effect of maternal depressive symptoms assessed during the third trimester of pregnancy on infant sleep problems (measured with the Children’s Sleep Habits Questionnaire – Infant Version) at 3 and 6 months postpartum (Castro Dias and Figueiredo, 2021). Lately, the China-Anhui Birth Cohort reported a negative effect of maternal prenatal depressive symptoms, only when comorbid with prenatal anxiety symptoms, on nocturnal sleep duration of 30-months olds (Ma et al., 2022). According to the foetal programming theory, programming signals, such as maternal depressive symptoms during pregnancy, are thought to influence the foetus’ organism, resulting in long-term repercussions, for instance on infant sleep (Davis et al., 2018; Castro Dias and Figueiredo, 2021). In addition, maternal cardio-metabolic conditions (such as GDM) also represent an important programming pathway, since prenatal maternal obesity is associated with toddler sleep problems (Mina et al., 2017).

Similarly to our results showing a prospective association between maternal depressive symptoms at 6–8 weeks postpartum and at one year postpartum, respectively with shorter infant night sleep duration and mothers considering the sleep of their infant as a very serious problem at 1 year postpartum, others also observed significant associations between these parameters in non-GDM samples (Armitage et al., 2009; Castro Dias and Figueiredo, 2021). Maternal depression assessed at 2 weeks postpartum was associated with longer infant nocturnal sleep latency and shorter infant sleep episodes measured by actigraphy, from 2 to 24 weeks postpartum (Armitage et al., 2009).

Similarly to our results showing a significant association between maternal depression and considering infant sleep at 1 year postpartum to be a serious problem, a study on mothers without GDM showed bidirectional associations between infant unsettled sleep and maternal depressive symptoms at 2 weeks, 3 and 6 months postpartum (Castro Dias and Figueiredo, 2021). According to a systematic review, compared to mothers without depressive symptoms, depressed mothers tend to adopt poorer maternal care toward their infant (e.g., lower sensitivity or emotional involvement; Slomian et al., 2019). Thus, maternal depressive symptoms may interfere with the mother-infant relationship and maternal perception of their infant (Slomian et al., 2019). As a result, this may lead to insensitive maternal night time behaviours that could prevent the infant from acquiring self-regulation competencies, which are necessary for optimal sleep development (Sadeh et al., 2010).

The social support provided via regular medical follow-ups during pregnancy may have had a carry-over effect and may have buffered the effect of maternal depression symptoms on some of the offspring’s behaviour, such as the number of night waking. Indeed, a study conducted on a similar population demonstrated a positive evolution of mental health that was hypothesised to be caused by medical social support (Gilbert et al., 2021). Additionally, in the current study, maternal social support (emotional and instrumental support) correlated inversely to number of infant night waking. Alternatively, evidence showed that women with GDM have higher depressive symptoms (e.g., anhedonia; Wilson et al., 2020), which have been associated with lower motivation to conduct physical activity (Carter and Swardfager, 2016). In previous studies, lower maternal physical activity during the perinatal period was associated with higher sleep problems in infants (Nakahara et al., 2021).

Regarding our *post hoc* analysis, results showed that even in mothers who perceived the sleep of their infant as “*not a problem at all*,” 34.69% of infants slept ≤ 11 h during the night (Iglowstein et al., 2003; Association Vaudoise d’Aide et de Soins à Domicile, 2019; Naître et grandir, 2019). Moreover, in infants sleeping ≤ 11 h per night, half of the mothers actually perceived infant sleep as not being a problem at all, thus showing a discrepancy between recommended night time sleep duration in infants and maternal perception of infant sleep. This may suggest that mothers perceive their infant sleep to be better than it actually is and, thus, may not have sufficient awareness of the necessary amount of nocturnal sleep one-year old infants need. The second observation suggests that there is a link between infant sleep duration and maternal perception of infant sleep as being a small to serious problem and that this could help to identify children at higher risk for insufficient sleep.

Raising awareness of infant sleep in mothers with GDM may be important to prevent potential adverse metabolic outcomes in their offspring. Indeed, early in life, infants with short sleep duration are at higher risk of obesity (Chen et al., 2008; Katzmarzyk et al., 2015; Miller et al., 2018). For instance, a meta-analysis demonstrated that for each hour increase in sleep in childhood and adolescence, the risk of overweight and obesity was reduced by 9% (Chen et al., 2008). Moreover, a negative association between sleep duration and the risk of hyperglycaemia was shown in children of mothers with GDM (Zhang et al., 2017), although this metabolic parameter may also be increased due to the fact that infants with lower sleep duration also experience obesity more frequently (Miller et al., 2018). Thus, with the assumption that sleep problems become chronic over the first year of life (Jenni et al., 2005), children of mothers with GDM who have a short nocturnal sleep duration may be at higher risk of developing subsequent hyperglycaemia and overweight/obesity. It is therefore of utmost importance to better understand the mechanisms involved in the association between infant nocturnal sleep duration and ensuing risk of developing metabolic issues.

### Limitations

The study has several limitations: First, the current data were derived from the control group of the *MySweetHeart trial* (Horsch et al., 2018). Although the control group received usual GDM care, participant bias cannot be excluded. In addition, participating in a trial on psychological and metabolic health may have affected the ecological validity of our study, although our mean levels of HbA1c and depression scores are similar to measures in a Swiss cohort of women with GDM (Gilbert et al., 2021). Second, a social desirability bias cannot be eliminated, as the data were self-reported. Also, maternal depressive symptoms may have influenced maternal perception of their infant. Third, the specific role of medical social support and physical activity in the associations between maternal depressive symptoms and infant sleep outcomes could not be investigated in the current study and may have impacted our results.Fourth, the current study contains up to 23.2% of missing data in infant sleep outcomes, although the percentage of missing data for the EPDS was only 1.1% (see Table 1). Nevertheless, given that this represents less than 30%, the power and validity of the results should not be impacted. *Post hoc* power indicators of significant associations range from 0.51 to 0.75, and in the adjusted model of 0.96. However, smaller *post hoc* power indices were found for some non-significant associations, suggesting probable type II errors. Therefore, these results would gain to be replicated with a larger sample size. Fifth, the present study would have benefited from comparing the current sample with a control group of women without GDM.

### Research and Clinical Implications

Future research analysing the associations between maternal depressive symptoms and infant sleep in GDM populations should (a) include actigraphy measurements of infant and maternal sleep and thus include maternal sleep as a potential mediator, as well as clinical interviews, (b) measure these variables at multiple time points during the perinatal period to test unidirectional and bidirectional associations, as previously suggested (Castro Dias and Figueiredo, 2021), and (c) assess additional potential confounding factors, such as the role of medical social support and physical activity. Furthermore, given that our sample seems to have a poor perception of the adequate amount of sleep infants require, we suggest raising awareness on infant sleep duration. We suggest a two-step approach. First, we recommend including psychoeducation on infant sleep at the first postpartum visit. Second, paediatricians and other health care professionals in infancy should specifically screen for infant sleep problems in GDM populations, even in the absence of maternal complaints.

## Conclusion

The current study examined the prospective associations between maternal depressive symptoms during and after pregnancy and infant sleep outcomes at 1 year postpartum in a GDM population. Maternal depressive symptoms at the first GDM visit and at 6–8 weeks postpartum were negatively and prospectively associated with infant nocturnal sleep duration. Furthermore, maternal depressive symptoms and maternal perception of infant sleep at 1 year postpartum were associated. We were able to partially confirm the prospective associations between maternal postpartum depressive symptoms and infant sleep in a GDM population. In addition, findings resulting from a *post hoc* analysis suggested misconceptions of mothers with GDM regarding the sufficient amount of nocturnal sleep needed for 1-year old infants. Since infants of mothers with GDM already have a higher risk of metabolic disorders and, given that sleep disruption augments that risk in the general population, this study strongly recommends strengthening psychoeducation regarding infant sleep for mothers with GDM and to particularly focus on women with depressive symptoms.

## Data Availability Statement

The raw data supporting the conclusions of this article will be made available by the authors, without undue reservation.

## Ethics Statement

The studies involving human participants were reviewed and approved by The Human Research Ethics Committee of the Canton de Vaud approved the study protocol (study number 2016-00745). The participants provided their written informed consent to participate in this study.

## Author Contributions

LG, VS, JP, and AH: conceptualization. LG, DQ, JP, and AH: methodology. LG: validation, formal analysis, and software. LG, VS, DQ, JP, and AH: writing – review and editing. LG, DQ, JP, and AH: project administration. LG and DQ: investigation and data curation. JP and AH: resources, supervision, and funding acquisition. LG and VS: writing – original draft preparation and visualization. All authors contributed to the article and approved the submitted version.

## Conflict of Interest

The authors declare that the research was conducted in the absence of any commercial or financial relationships that could be construed as a potential conflict of interest.

## Publisher’s Note

All claims expressed in this article are solely those of the authors and do not necessarily represent those of their affiliated organizations, or those of the publisher, the editors and the reviewers. Any product that may be evaluated in this article, or claim that may be made by its manufacturer, is not guaranteed or endorsed by the publisher.
